# Digital Dentistry: Advances and Challenges

**DOI:** 10.3390/jcm9124005

**Published:** 2020-12-11

**Authors:** Falk Schwendicke

**Affiliations:** Department of Oral Diagnostics, Digital Health and Health Services Research, Charité-Universitätsmedizin, 14197 Berlin, Germany; falk.schwendicke@charite.de

Dental diseases like caries or periodontitis are among the most prevalent in the world, generating significant subjective and financial burden to individuals and healthcare systems. For detecting and managing dental diseases, dentists increasingly rely on digital technologies. With the explosion in data amount and availability and the technical means to effectively lever them, this trend towards digital dentistry is accelerating: Digital dentistry is nowadays much more than computer-aided design and manufacturing (CAD-CAM), it entails diagnostics, decision-making, treatment conduct and re-evaluation as well as lifelong management of patients’ oral health. We are fast moving towards workflows which are truly digital beyond only the provision of dental restorations or implants but permeate into every aspect of our profession.

A major element in these advances is Artificial Intelligence (AI). AI is already included in various dental and dental technology workflows, including and prominent in the already mentioned CAD-CAM area (here closely interwoven with innovations in the field of imaging). The next task will, however, be to make better use of this technology which in turn itself will make better use of data, e.g., general and dental history, clinical data, various image and further diagnostics data which are currently assessed, stored and used in isolation, but not really employed to their full potential: Individual dentists are not fully able to integrate all these data at their disposal, and hence use only a fraction of it for therapy planning and treatment. As in many other areas of the healthcare system, this is mainly due to the fact that these data are often collected in an unstructured way and stored in disconnected data silos, but also as humans struggle to “compute” such vast amounts of data in parallel.

Synthesizing these data will allow illumination of a path towards a more predictive, personalized, preventive and participatory dentistry (P4-dentistry), if all goes well. This if, however, is a big one: With all excitement, current applications and research findings suffer from a large range of limitations, and many studies in this emerging field do not fully adhere to established standards of rigorous planning, conducting and reporting demanded by evidence-based research practice. So far, digital dentistry is essentially facing a schisma; industry-driven technological solutions (mainly in the CAD-CAM and imaging sector) are already reality, while AI-technologies focusing on diagnostics and treatment planning are rather concepts than robust applications at present. Bridging that schisma will be one major task of the future! There is a high need to bring technology and dental medicine together; joint and interdisciplinary research, research groups and research initiatives are needed to overcome this split and to nurture this promising field.

The present Special Issue of the Journal of Clinical Medicine is dedicated to Digital Dentistry in all its wealth, but also its limitations. May it help to overcome the latter!

